# Supplementary material for: Estimation method for impact force source in thick coal seams and its application in rockburst risk quantification
Source: PLoS One. 2025 Jun 6;20(6):e0325396. doi: 10.1371/journal.pone.0325396 (PMC12143892; doi:10.1371/journal.pone.0325396)
Supplement: Attachment — Submitted filename: Response_to_Reviewer_Comments_20250407.docx [file pone.0325396.s002.docx]

**Response to Reviewer Comments and Manuscript Revision**

**Manuscript ID**: PONE-D-25-09536

**Title**: Study on Estimation Method for Coal Burst Force Sources in Extremely Thick Coal Seams and Quantitative Evaluation of Coal Burst Risk

**Corresponding Author**: ZHU Quanjie

Dear Editor and Reviewers,

Thank you for your letter and the reviewers' constructive comments on our manuscript. We have carefully addressed all concerns and revised the manuscript accordingly. All modifications in the revised manuscript are highlighted for easy identification. Below we provide point-by-point responses to the reviewers' comments.

We believe these revisions have significantly improved the manuscript and hope it now meets the journal's standards. Thank you again for your valuable feedback. Please do not hesitate to contact us if further modifications are required.

Thank you for your time and consideration. We look forward to your feedback and to the continued evaluation of our manuscript.

Kind regards,

Quanjie ZHU

zhqj2016@ncist.edu.cn

**Reviewer #1: Your manuscript presents a comprehensive study on estimating coal burst force sources in extremely thick coal seams beneath massive gravel rock. However, enhancing the clarity of the methodology, broadening the discussion's practical context, and providing further justification for certain methodological choices would strengthen the paper.**

**1、 Explicitly list the key assumptions made in the stress estimation models (e.g., load distribution assumptions, boundary conditions). This would help readers better understand the limitations and applicability of your models.**

**Reply:** Thank you for the expert’s valuable comments. When establishing the static support pressure estimation model for strike/dip under insufficient mining conditions, the key assumptions, theoretical basis, and limitations are as follows:

(1) In terms of load distribution, two assumptions are introduced: the dominant key layer assumption and the uniform load simplification. The former assumes that only the key layer bears the overlying rock structure load, while the self-weight of the non-key layer is converted into a uniform load (q = γh). This is based on the key layer theory, which confirms that thick hard rock layers control over 90% of the load transfer. The latter is based on St. Venant's principle (applicable when the stress influence range is greater than three times the layer thickness), and it mainly considers that the load of the non-key layer acts uniformly on the underlying key layer. The limitations of these assumptions are as follows: The first assumption neglects the rheological effect of weak interlayers (which may lead to overestimating the fracture angle α) and does not account for the local load redistribution caused by the formation of the coal seam’s plastic zone. The second assumption becomes limited when extreme dip angles (>25°) lead to significant load eccentricity. Furthermore, it is not applicable to thin bedrock conditions (<5 times the mining height).

(2) In terms of boundary condition assumptions, linearization of the fracture line and static stress field assumptions are introduced. The former assumes that the line connecting the front end of each key layer’s delamination is simplified to a straight line (with a constant fracture angle α), which is consistent with field conditions and results from similar simulation experiments. The limitation of this assumption is that it does not consider the stepped fractures that may occur when there is a sudden change in gravel layer thickness (requiring the introduction of a segmented angle correction) and the time effect on the fracture angle under dynamic mining conditions (α may decrease by 5-8°). The latter assumption neglects the dynamic disturbance caused by mining speed. This assumption is valid when the mining face recovery rate is less than 5 m/day, but when the advance speed exceeds 10 m/day, the dynamic load factor and the transient impact effects caused by periodic compression need to be considered.

(3) In terms of layer behavior, the assumption of ideal delamination behavior and fracture angle consistency is introduced. According to elastic plate theory, once the key layer delaminates, it completely loses its ability to transmit horizontal stress, and the massive gravel rock may exhibit a cantilever beam effect. According to isotropic rock body statistics, the strike and dip fracture angles are the same. However, when dominant joint sets exist, anisotropic effects need to be considered.

As can be seen, these assumptions essentially construct a theoretical framework of "quasi-static, layered isotropy, and ideal fractures." Under simple geological conditions (single key layer, low tectonic stress), this method can meet the needs of impact risk assessment.

**2、 While the discussion section touches on future research directions, a more detailed discussion on the limitations of the current approach (e.g., uncertainty in tectonic stress quantification) would be valuable.**

**Reply:** We fully agree with the reviewer’s important comment on the uncertainty of tectonic stress quantification.

The current methods for quantifying tectonic stress do have certain limitations, primarily due to factors such as errors from simplified empirical assumptions, rough parameter values, and the absence of dynamic effects. Although these methods have some limitations in terms of precision, the advantage of using empirical simplifications to quickly estimate tectonic stress remains quite significant from the application perspective.

As discussed in Section 5.2, the current empirical model indeed faces the issue of high parameter sensitivity. This highlights the core direction for future research. We are developing a probabilistic evaluation module based on the fault slip potential theory, which incorporates the uncertainty of fault geometric parameters into Monte Carlo simulations. This module is expected to reduce the prediction confidence interval of the stress increment coefficient (*k*) and the impact range (*L*).

**3、 Consider adding more detailed captions and ensuring that all axes, scales, and legends are clearly explained.**

**Reply:** Thank you for the expert's comments. Based on the expert's suggestions, we have made revisions and improvements to the relevant figures in order to meet the journal's requirements.

**4、 While the introduction explains the significance of the study, it could benefit from more in-depth references to recent research in the past five years. Highlight gaps in the literature this study addresses, such as: compgeo.2024.106095; tafmec.2024.104691; fuel.2023.129584**

**Reply:** Thank you for the expert's feedback. As requested, we have added the relevant references and emphasized the innovative value of the research content as well as its significance in the field. Please refer to the references section in the revised manuscript for details.

**5、 Emphasize how the quantitative approach can bridge the gap between theoretical predictions and on-site applications in complex geological settings.**

**Reply:** The core value of quantitative methods in coal burst prediction lies in transforming theoretical mechanics principles into engineering-based evaluation tools through mathematical modeling and data-driven approaches.

This study, through a triple quantitative strategy—parameter explicitness, model dynamicization, and indicator standardization—achieves for the first time the 'computational' transformation of coal burst theoretical models under complex geological conditions. On-site verification demonstrates that this method improves the alignment between theoretical predictions and engineering decisions, providing a reusable technical paradigm for risk prevention and control in deep mining.

**6、 Highlight avenues for future research, such as addressing the challenges of non-linear interactions between different stress sources.**

**Reply:** Although the current study has achieved a linearized approximation of multiple stress sources, the nonlinear interactions between different stress sources (such as the synergistic abrupt change between fault activation and mining disturbance) remain an unsolved issue. In the future, we will explore this further from multiple aspects, including: ① Multi-scale constitutive theory: integrating Discrete Element Method (DEM) and Finite Element Method (FEM) to depict the full chain response from rock mass fracture to regional stress redistribution; ② Metaverse simulation platform: developing a virtual-physical interactive digital twin system for mine stress fields that supports real-time nonlinear predictions. These breakthroughs will enable the theoretical model to truly adapt to the extremely complex environment of deep mining.

**7、 Strengthen your discussion by comparing your method not only with traditional qualitative approaches but also with emerging quantitative methods.**

**Reply:** Compared to traditional empirical methods, this study advances the prediction of rockburst pressure from "phenomenon description" to "mechanism calculation" through stress source decoupling quantification and dynamic parameter inversion. In contrast to purely data-driven methods, the physical core of this approach ensures robustness in data-scarce scenarios.

Traditional theoretical estimation and field measurement methods still have cost advantages under simple geological conditions (e.g., shallow, single coal seams), but their static characteristics and low resolution are inadequate for meeting the demands of deep and complex mining operations. The quantitative method in this paper achieves a leap from "qualitative empirical judgment" to "quantitative dynamic early warning" through three innovations: ① stress source decoupling technology; ② microseismic-stress collaborative inversion; ③ dynamic risk index.

**8、 The comparison with existing coal burst risk evaluation methods is a strength. Expanding this section to discuss potential scenarios where your method performs particularly well (or less well) compared to conventional methods could further highlight its advantages.**

**Reply:** Existing coal burst risk assessment methods primarily focus on the analysis of the physical and mechanical properties of the coal seam itself, mining disturbances, and stress distribution. These methods largely concentrate on the study of the coal seam itself, while neglecting the impact of the overlying strata (such as massive gravel rock), leading to potentially inaccurate results in specific scenarios.

In contrast, the method presented in this paper integrates the stress superposition effect under extremely thick coal seams with massive gravel rock, using a combination of microseismic monitoring and UDEC numerical simulation technologies. The "superimposed stress estimation" approach is employed to quantify the coal burst risk. In specific scenarios, especially when dealing with the special overlying conditions of massive gravel rock, the method proposed in this paper allows for a more precise simulation of the stress interaction between the coal seam and overlying rock layers, overcoming the limitations of traditional methods under complex geological conditions. Specifically, the advantages are:

By combining stress calculation, microseismic monitoring, and numerical simulation, the method can comprehensively assess the interaction between the coal seam and overlying rock layers, providing a more comprehensive risk assessment.

The "superimposed stress estimation" method can accurately estimate stress distribution and accumulation at different levels, improving the predictive ability for coal burst risks.

However, in certain specific scenarios, such as unconventional mining areas or extremely complex geological conditions, the assumptions of the model and the accuracy of input data may affect the final risk assessment results. In such cases, it is necessary to continuously refine the model through field measurement data to ensure the reliability of the prediction results.

In conclusion, the method presented in this paper offers a new approach for coal burst risk assessment and demonstrates strong application potential in specific complex mining areas. However, further verification and optimization in multiple scenarios are needed.

**Reviewer #2:**

**1- The title should be improved.**

**Reply:** Thank you for the expert's comments. Based on the expert's suggestions, we have revised the original title to "Method for Estimating the Impact Force Source in Thick Coal Seams and Its Application in Quantitative Evaluation of Impact Risk," combining the impact force source estimation method and impact risk evaluation to better align with the accurate expression of the paper.

**2- The objectives and the rationale of the study are recommended to be clearly stated.**

**Reply:** Thank you for your suggestion! The main objective of this study is to clarify the occurrence mechanism of rockburst in thick coal seams under giant-thick conglomerates, and to explore a method for estimating the impact force source and a quantitative evaluation approach for impact risk in thick coal seams under giant-thick conglomerates. Theoretically, the study is based on elastoplastic mechanics, rock mechanics, and the theory of overlying rock spatial structures. It focuses on revealing the complex stress transfer mechanism between the giant-thick conglomerates and the coal seam, establishing a stress superposition estimation model, and integrating microseismic monitoring and UDEC numerical simulation technologies to achieve recognition and early warning of coal mine rockburst risks, thus providing reliable theoretical guidance and technical support for mine safety production.

**3- The concluding remarks of the abstract are not well-written. It's merely the repetition of the objectives and title of the manuscript. Please add method limitations and justification to the abstract.**

**Reply:** Thank you for your suggestion! In the conclusion section of the abstract, we will further supplement the limitations of the research methods and the supporting arguments based on the objectives and title. Specifically, although this study explores the impact burst mechanism and early warning methods through various means such as stress superposition theory, microseismic monitoring, and numerical simulation, there are still certain limitations in practical applications. For example, the applicability of some theoretical models may be affected by the actual conditions of the mine and the characteristics of the overlying rock layers. Additionally, the accuracy and response time of microseismic monitoring technology may have some errors. Therefore, further experimental verification and field application studies are important steps in validating the effectiveness of the methods. These additions will make the conclusion more comprehensive and strengthen the argument of the research.

**4- The innovation of using this study is not very clear. I do not see a clear reason that this study can perform better than others. Why did the authors choose the method for this study?**

**Reply:** Thank you for your valuable suggestion! In the section on the innovation of the study, we will further clarify the unique advantages of this method and compare it with existing research. Specifically, this study proposes a new method for estimating impact force sources and a quantitative evaluation method for impact risk by combining the stress superposition theory under giant-thick conglomerates and microseismic monitoring technology. Compared to traditional methods, our approach can more accurately estimate the stress distribution in coal seams caused by the stress superposition from the overlying rock layers. Additionally, it integrates field measurement data and numerical simulations, improving the timeliness and accuracy of impact risk assessment. Furthermore, this method shows strong applicability in thick coal seams and complex geological conditions, addressing the limitations of traditional methods under these special circumstances. Therefore, we believe this method has significant innovation and practical value in impact burst early warning and risk evaluation.

**5- The necessity & novelty of the manuscript should be presented and stressed in the "Introduction" section.**

**Reply:** Thank you for your suggestion! We will further emphasize the necessity and novelty of this research in the introduction. Specifically, during the current coal mining process, especially under thick coal seams and complex geological conditions, rockburst incidents frequently occur, posing a significant challenge to mine safety. However, existing studies mainly focus on the mechanical properties and stress analysis of coal seams themselves, overlooking the impact of giant-thick conglomerate layers on the stress transfer in coal seams. As a result, current early warning methods struggle to address rockburst issues under complex geological conditions. Therefore, this study introduces the stress superposition theory, combined with microseismic monitoring and numerical simulation, to propose a new method for analyzing the mechanisms of rockbursts and early warning for thick coal seams under giant-thick conglomerates. This research fills this gap, providing a highly novel and practical approach. Through this study, more precise risk assessments and early warning support for mine safety can be provided, holding significant theoretical and practical value.

**6- The application/theory/method/study reported is not in sufficient detail to allow for its replicability and/or reproducibility. Therefore, it is suggested to make it clear to show all steps to build the model.**

**Reply:** Thank you for your suggestion! We will further elaborate on the steps of model construction in the methods section to ensure the reproducibility of the study. Specifically, as detailed in the section "**2 Impact Force Source Estimation Model for Extremely Thick Coal Seams**," we describe the model construction process in detail, including the selection of input data, model assumptions, parameter settings, and the calculation methods for each step. In addition, we provide the corresponding mathematical formulas, calculation formulas, and model verification process to ensure that other researchers can replicate the same results based on our description. At the same time, we will clearly state the assumptions and limitations of the model to enhance the transparency and credibility of the research.

**7- The problem statement and gap study are not clear.**

**Reply:** Thank you for your feedback! We will further clarify the problem statement and research gaps in the paper. Specifically, we will more clearly outline the shortcomings in the current research, particularly the gaps in the study of the occurrence mechanisms and predictive warning methods of coal burst under extremely thick coal seams covered by massive gravel rock. Additionally, we will highlight the unresolved issues in existing studies, such as the stress transfer mechanism and the impact of gravel layers on coal seams, and clearly present the gaps that this study fills and its innovations. This will help to better demonstrate the significance and necessity of our research. As added in the introduction: Existing research largely focuses on the mechanical properties and stress analysis of coal seams themselves, neglecting the influence of massive gravel rock layers on stress transfer in coal seams, which makes existing warning methods inadequate for addressing coal burst issues under complex geological conditions. Therefore, this study introduces stress superposition theory, combines microseismic monitoring and numerical simulation, and proposes a method for analyzing the occurrence mechanisms and early warning of coal burst under extremely thick coal seams covered by massive gravel rock. This fills the research gap and is highly novel and practical. This study provides more accurate risk assessment and early warning support for mine safety production, with significant theoretical and practical value.

**8- The method is not clear. Therefore, it must be shown and clarified to show all steps.**

Reply: Thank you for your valuable comments! We will further improve and clearly present all the research steps in the methodology section to ensure that each part of the process is logically clear and easy to understand. Specifically, we will provide a detailed description of the model construction process, including the mathematical derivations of each step, the assumptions, parameter selection, the numerical simulation process, and the related calculation methods. This will ensure that readers can fully understand the implementation of the research and be able to replicate our study. This approach will not only enhance the transparency of the methodology but also contribute to further validating the reliability and effectiveness of the research.

**9- The interpretation of results and study conclusions are not supported by providing the reasons behind why they show that. Therefore, it is recommended to deepen the discussion.**

**Reply:** Thank you for your valuable suggestion! We will further deepen the analysis in the results interpretation and conclusions sections, especially by providing a detailed discussion of the underlying causes of the research findings. In the revision, we will focus on the study of "the occurrence mechanism and risk assessment method of coal burst under extremely thick coal seams covered by massive gravel rock," with "impact source estimation" as the core. We will analyze the possible physical mechanisms and influencing factors, discuss the potential variables that affect the research results, and provide an in-depth explanation of the advantages and limitations of our method, comparing it with existing literature. Additionally, we will explore the significance of the research results in practical applications, in order to provide more theoretical support and practical guidance for future research and practice.

**10- It is recommended to emphasize the strengths of the study clearly.**

Response: Thank you for your suggestions! In the revision, we will clearly highlight the advantages of this study, especially the innovation, applicability, and uniqueness of the method compared to existing research. We will thoroughly explain the advantages of the proposed method in coal burst monitoring and early warning under the conditions of extremely thick coal seams and massive gravel rock, and how it improves the accuracy and efficiency of early warning. Additionally, we will compare this method with traditional methods to emphasize the advantages of this study in stress estimation, microseismic monitoring, numerical simulation, and other aspects. Furthermore, we will emphasize the practical application prospects of this method, especially its contribution to enhancing safety in coal mining operations.

**11- The limitations of the study should be stated.**

**Response:** Thank you for your valuable comments! In the revision, we will add a section on the limitations of the study. Although the coal burst monitoring and early warning method proposed in this study has good application prospects under the conditions of extremely thick coal seams and massive gravel rock, there are still some limitations. First, the applicability of the research method may be constrained by the geological conditions of the mining area, and model parameters may need to be adjusted in different regions or complex geological structures. Secondly, the microseismic monitoring technology and stress estimation methods depend on the accuracy of data collection and the rationality of sensor placement, which may be limited in the practical application of some mining areas. Finally, discrepancies between numerical simulation results and real-world conditions may also affect the accuracy of the model. Therefore, in future research, we will further validate and optimize this method in order to improve its general applicability and precision.

**12- The manuscript structure, flow, or writing needs some improvements.**

**Response:** Thank you for your valuable comments! We will improve the structure and logic of the manuscript to enhance the clarity and flow of the article. During the revision, we will further optimize the chapter arrangement to make the content more organized, ensuring better connections between sections. At the same time, we will adjust the expression of certain paragraphs to make the language more concise and accurate, in order to better convey the core ideas and innovations of the research. Additionally, we will adjust and strengthen the logical relationships of some content to make the article more rigorous and persuasive. Thank you for your feedback, which will help us further improve the quality of the manuscript!

**13- The manuscript is benefit from language editing. The English of the paper is readable; however, I would suggest the authors to have it checked preferably by a native English-speaking person to avoid any mistakes.**

**Response:** Thank you for your suggestion! We will follow your advice and have the manuscript polished by a native English speaker to ensure the language is more fluent, natural, and meets academic writing standards. This will help improve the language quality and readability of the article, ensuring that the research findings are communicated clearly and accurately to the international academic community. Thank you again for your valuable feedback!

**14- I noticed that the conclusion section tends to repeat the abstract and results. The conclusion paragraph should be short, impactful, and direct the reader to this research's next steps and opportunities.**

**Response:** Thank you for your feedback! We will revise the conclusion section to avoid repetition with the abstract and results. The conclusion will be more concise and impactful, clearly summarizing the core findings of the study and discussing the limitations and future research directions, to provide clear guidance for future research. Thank you for your valuable comments!

**15- It will be nice to add some new references to show that your study is updated, such as:**

Zhou, Zhanxin, and Ruibo Wu. "Stock Price Prediction Model Based on Convolutional Neural Networks." Journal of Industrial Engineering and Applied Science 2.4 (2024): 1-7;

Alakbari, Fahd Saeed, et al. "Prediction of critical total drawdown in sand production from gas wells: Machine learning approach." The Canadian Journal of Chemical Engineering 101.5 (2023): [2493-2509](tel:2493-2509).;

Alakbari, Fahd Saeed, et al. "Deep learning approach for robust prediction of reservoir bubble point pressure." ACS omega 6.33 (2021): [21499-21513](tel:21499-21513).;

Alakbari, Fahd Saeed, et al. "A gated recurrent unit model to predict Poisson's ratio using deep learning." Journal of Rock Mechanics and Geotechnical Engineering 16.1 (2024): 123-135;

Zhou, Zhanxin, and Ruibo Wu. "Stock Price Prediction Model Based on Convolutional Neural Networks." Journal of Industrial Engineering and Applied Science 2.4 (2024): 1-7;

Wu, Ruibo, Tao Zhang, and Feng Xu. "Cross-Market Arbitrage Strategies Based on Deep Learning." Academic Journal of Sociology and Management 2.4 (2024): 20-26.

Alakbari, Fahd Saeed, et al. "Prediction of critical total drawdown in sand production from gas wells: Machine learning approach." The Canadian Journal of Chemical Engineering 101.5 (2023): [2493-2509](tel:2493-2509).

Hassan, Anas M., et al. "A new insight into smart water assisted foam SWAF technology in carbonate rocks using artificial neural networks ANNs." Offshore Technology Conference Asia. OTC, 2022.

**Response:** Thank you for your suggestion! We will add the latest references in the relevant fields, particularly in machine learning and rock mechanics, to enhance the timeliness and academic depth of the paper. By citing these references, we will further strengthen the research background and ensure that the literature review covers the latest advancements in the field. Thank you again for your valuable feedback!

**Reviewer #3:**

**Dear Authors,**

**Thank you for submitting your manuscript, "Study on Estimation Method for Coal Burst Force Sources in Extremely Thick Coal Seams and Quantitative Evaluation of Coal Burst Risk," to PLOS ONE. Your research tackles a pressing challenge in underground mining safety—preventing coal bursts in thick coal seams—and your work at Qianqiu Coal Mine in Henan, China, offers valuable insights. The combination of stress estimation, microseismic monitoring, numerical modeling, and field validation is impressive, and your focus on enhancing mine safety is both timely and impactful. To help strengthen your manuscript and maximize its contribution to the field, I’d like to offer some suggestions for improvement.**

**1. Introduction**

**1.1. What Are "Extremely Thick Coal Seams"?**

**In the introduction, please specify what "extremely thick" means in your study—perhaps a thickness range (e.g., >10 meters?)—so readers can better understand the context of your work.**

**Response:** Thank you for your valuable suggestions! We will follow your advice and clarify the definition of "extremely thick coal seams" in the paper. For example, extremely thick coal seams typically refer to seams with a thickness greater than 10 meters, and we will provide a detailed definition based on the actual conditions of the mine to ensure clear understanding of this concept by the readers.

**1.2. Simplify Technical Descriptions**

**The sections on stress estimation and microseismic monitoring are detailed but dense. A simpler explanation or a concise summary of these methods would improve readability while keeping the technical depth intact.**

**Response:** Thank you for your suggestion on simplifying the technical descriptions of stress estimation and microseismic monitoring. We understand that the technical details of stress estimation and microseismic monitoring may lead to confusion among readers. In the revision, we will simplify the relevant sections, focusing on the core ideas and application steps of the methods while reducing excessive technical details. Specifically, we will improve the following aspects: 1) streamline the theoretical background of stress estimation, focusing directly on the establishment and application of the estimation model; 2) simplify the description of microseismic monitoring, emphasizing the connection between key parameters (such as fracture angle and strike span) and actual monitoring. Thank you again for your valuable suggestion, and we will fully consider these comments in the revised manuscript.

**2. Methodology**

**2.1. Quantifying Tectonic Stress**

**Tectonic stress plays a big role in coal bursts, but your study treats it in a semi-quantitative way. Could you include more precise measurements, perhaps using geophysical data or advanced models, to quantify its impact?**

**Response:** Thank you for the valuable suggestion! We fully agree that geophysical data has important advantages in quantifying tectonic stress. While our current approach mainly relies on stress estimation models and microseismic monitoring, the introduction of geophysical data will undoubtedly improve the accuracy of tectonic stress quantification. Therefore, in future research, we will try to incorporate geophysical data from actual mines, combined with microseismic monitoring results, to further validate the accuracy of tectonic stress. We will add a discussion of this issue in the revision and suggest that future research could combine geophysical data to further improve the model. Thank you for your useful suggestion!

**2.2. Why These Microseismic Parameters?**

**You use fracture angles and strike spans from microseismic data to assess coal burst risk. Could you explain why these specific parameters were chosen and how they predict bursts?**

**Response:** Thank you for your valuable comments. Regarding the selection criteria for microseismic parameters (fracture angle, strike span), we chose these parameters because they are important physical indicators for describing the fracturing of rock layers and the stress distribution in coal seams. The role of microseismic monitoring is not only as an independent monitoring tool but also closely related to the shock pressure source estimation method.

Specifically, when rock layers are subjected to stress, they undergo fractures or minor breaks, which are accompanied by the generation of microseismic signals. Through the array of microseismic sensors we deployed, we can capture these vibration signals in real-time, and by analyzing the time, frequency, and spatial distribution of the signals, we can pinpoint the location of the fractures and the extent of crack propagation. By analyzing the propagation path and energy distribution of the vibration signals, we can estimate the fracture angle (fracture angle) and strike span. These parameters provide foundational data for the shock pressure estimation model in this study. With the spatial localization characteristics of microseismic monitoring technology, we can accurately determine key parameters like fracture angle (α) and strike span (L). These parameters form the foundation for the stress estimation model proposed in section 2.1, "Stress Estimation Method for Extremely Thick Gravel Rock."

Therefore, microseismic monitoring results not only provide real-time monitoring and capture of rock layer fracture signals, helping to identify potential shock pressure zones, but also contribute to the establishment of coal seam stress estimation models through accurate fracture angle and strike span data.

**2.3. Comparing to Other Models**

**Your new risk evaluation model is exciting, but how does it stack up against existing tools like BurstRisk? A direct comparison would highlight what makes your approach stand out.**

**Response:** Thank you for your valuable suggestion. Indeed, existing shock pressure risk assessment tools, such as the BurstRisk model, have been widely used in mine safety research. Compared with these existing tools, our research has some significant innovations. First, the existing BurstRisk model primarily relies on traditional stress analysis and coal seam physical property assessments, while our method combines microseismic monitoring technology and stress estimation models, capturing spatial information of rock layer fractures and the fracturing process using microseismic signals, estimating key parameters such as fracture angle and strike span, and integrating the characteristics of extremely thick gravel layers to conduct in-depth research into the shock pressure mechanism. This innovative combination provides more precise data support for early warning of shock pressure in mines.

Additionally, our research introduces a shock risk assessment method based on stress estimation, combined with microseismic monitoring and numerical simulation technology, enabling real-time assessment of risk zones in mines, rather than relying solely on static models. This makes our assessment method more practical in dynamic monitoring and real-time response. We will further emphasize these innovations in the manuscript and provide a detailed comparison with existing models to highlight the uniqueness and advantages of our method. Thank you again for your suggestions; we will further elaborate on this comparison analysis in the revised manuscript.

**3. Results**

**3.1. Your work fits into a global effort to improve mine safety. Comparing it to studies like the one on roadway rockbursts in deep coal seams (e.g., this study) would show how your research builds on or differs from what’s out there.**

Response: Thank you for your insightful suggestion. We recognize the importance of positioning this research in the context of international studies. Therefore, we will supplement the manuscript with references to representative international literature on rock burst in deep coal seam tunnels.

These documents not only provide a theoretical foundation for this study but also indicate that current international research primarily focuses on the mechanical response and fracture evolution of coal seams, while there is relatively limited exploration into the stress coupling mechanisms between ultra-thick coal seams and their overlying giant-thick conglomerate strata. Therefore, this study combines microseismic parameters to deduce fracture characteristics and establishes a force-source estimation model, offering valuable insights into this research gap.

We will incorporate the above literature citations in the introduction and relevant sections, further highlighting the innovativeness and research value of this study in the global field of coal mine rockburst prevention and control. Thank you once again for your suggestions.

**3.2. Since you’ve developed a quantitative risk evaluation method, explaining how it improves on semi-quantitative systems (like BurstRisk) would strengthen your case.**

**Response:** We appreciate the reviewer's suggestion. We will supplement the comparative analysis of quantitative versus semi-quantitative systems. Compared to traditional semi-quantitative risk assessment methods (e.g., scoring systems, empirical classification), the proposed quantitative rockburst evaluation method directly quantifies stress sources through microseismic parameter inversion and stress estimation modeling, enabling more precise characterization of coal-rock system stress states and hazard distribution. Key advantages include:

(1) Enhanced scientific rigor: The stress superposition model, quantitative tectonic stress calculation, and microseismic inversion objectively reflect actual stress conditions, minimizing subjective bias;

(2) Sharper zoning: Enables quantitative classification and visualization of rockburst risks across different mining panels;

(3) Improved dynamic response: Integrates real-time microseismic monitoring for dynamic hazard trend tracking and early warning.

We will expand this discussion in the revised manuscript and strengthen comparative analyses with semi-quantitative methods in the Results & Discussion section to better demonstrate the methodological advantages. Thank you for this constructive input.

**3.3. If possible, include datasets like microseismic readings or stress measurements as supplementary files. This would let others verify or build on your work.**

**Response:** We thank the reviewer for this suggestion. We will consider making relevant microseismic and stress measurement datasets publicly available to facilitate verification and ensure reproducibility.

**3.4. If you used proprietary tools or data, consider suggesting open-source alternatives or other ways readers could replicate your results.**

**Response:** We acknowledge the reviewer's valid concern regarding reproducibility with proprietary software (UDEC for fracture angle simulation and stress modeling). In revision, we will:

(1) Specify all critical parameters (boundary conditions, constitutive models, meshing criteria);

(2) Provide pseudocode for core algorithms to enable replication using open-source platforms (FLAC3D, RS2, or FEM/DEM frameworks);

(3) Detail microseismic processing logic and recommend open-source alternatives (e.g., ObsPy).

Selected input data and model schematics will be included in appendices to enhance verifiability. We appreciate this suggestion to improve methodological transparency.

**4. Practical Applications and Limitations**

**4.1. You mention pre-cracking massive gravel rock to reduce burst risks—could you give more details on practical techniques or tools that could make this happen?**

**Response:** We thank the reviewer for highlighting this important application. Pre-splitting techniques are particularly effective for reducing stiffness and stress concentration in massive, homogeneous conglomerate strata. Key engineering approaches include:

(1) Directional deep-hole blasting: Controlled explosive fracturing along bedrock dip;

(2) Hydraulic fracturing: High-pressure fluid injection to create weak planes;

(3) Grouting modification: Borehole-based softening to induce structural heterogeneity.

These require 3D stress field modeling for optimal placement and microseismic monitoring for efficacy validation. As this study focuses on risk zoning rather than engineering implementation, we regret being unable to expand further due to length constraints. We appreciate this expert suggestion.

**4.2. Adding a section on your study’s limitations (e.g., assumptions in your stress models or challenges with tectonic stress) would give a fuller picture and point the way for future work.**

**Response:** We will add a dedicated "Limitations" section addressing: Simplifications in linear elastic theory and overburden structure modeling; Uncertainties in tectonic stress quantification due to data resolution; Microseismic parameter constraints from monitoring network precision.

These will be objectively evaluated to demonstrate academic rigor. Thank you for this valuable recommendation.

**5. Better Visuals**

**5.1. Diagrams like stress maps or risk zones would make your findings easier to grasp.**

**Response:** Thank you for the valuable feedback. We will enhance the paper by including stress distribution diagrams, risk zoning maps, and other visual aids to help readers better understand the research findings and conclusions.

**5.2. A table summarizing key results—say, how different stress factors contribute or how your predictions match real data—would help readers quickly see your main points.**

**Response:** We appreciate the reviewer’s detailed suggestion. In the revised manuscript, we will supplement a tabular summary of key results, particularly regarding the contribution analysis of stress factors. This table will quantify and compare the proportions and influence levels of conglomerate-transmitted stress, overburden self-weight stress, tectonic stress, and mining-induced stress in the total stress superposition. This will improve the clarity and comparability of the results, helping readers understand the relative roles of different stress sources in rockburst risk. Additionally, we will include relevant data sources and analytical methods to ensure the table is scientifically grounded and reproducible. Thank you once again for your constructive comments.

**Reviewer #4:**

**1. In the Introduction section, rockbursts phenomenon is described in general terms focusing on potential hazards. However, the rationale for the study is expressed through subjective judgments about the importance of the quantitative estimation approach/method. Instead of discussing the weaknesses of alternative approaches/methods, the authors focused on presenting the potential benefits of their proposed method. So noone will know about the rationale need for using quantitative estimation method.**

**Although the aim of the study is presented in sufficient detail, due to the lack of a discussion on the rationale makes Introduction can be accepted absent.**

**Response:** We sincerely appreciate the reviewer's constructive suggestion. In the revised manuscript, we have enhanced the introduction by:

Elaborating on the suddenness and high-energy release characteristics of rockbursts under ultra-thick coal seam and complex geological conditions

Systematically analyzing limitations of conventional semi-quantitative/empirical methods (e.g., rockburst propensity classification, coal energy index), particularly their subjectivity, poor adaptability, and low prediction accuracy

Emphasizing the critical need for quantitative stress estimation and risk assessment methods in high-stress, ultra-thick coal seam environments to improve monitoring and early warning precision

These modifications better justify the scientific significance and technical advantages of our proposed methodology.

**2. In the study frequent refereneces to previous/other reserches on the subject (e.g., “experts and scholars have conducted extensive research and field practices,” L573–574), these studies are not properly cited.**

**Response:** We thank the reviewer for identifying this oversight. We have:

- Removed all generic statements about "extensive studies"
- Added specific citations to support each referenced research finding
- Ensured all literature references comply with journal formatting guidelines
- The corrections have been implemented throughout the manuscript.

**3. Some of the approaches used in calculating effective stresses appear to be based on general geometry-weight relationships (stress calcualtions), while others seem to be constructed based on the authors’ assumptions. The rationale for using these assumption-based formulations is not clearly stated. Consequently, due to the calculations they are based on, it can be argued that the study lacks a solid theoretical foundation.**

**Response:** We appreciate this important technical comment. In revision, we have:

- Clearly delineated the theoretical foundations for both geometric-weight calculations and necessary assumptions
- Added supporting references and empirical validations for all modeling assumptions
- Explicitly stated each method's applicability boundaries
- While acknowledging the model's current limitations for rapid stress estimation at engineering scales, we have positioned this as groundwork for future improvements through multi-source data fusion and transparent geology approaches. The enhanced theoretical framework appears in Sections 2.1-2.3.

**4.The manuscript appears to be an attempt to merge two different and largely unrelated topics. The title, “Study on Estimation Method for Coal Burst Force Sources in Extremely Thick Coal Seams and Quantitative Evaluation,” suggests that the primary focus is on the estimation of coal burst forces. However, the Conclsuions section presents only the findings from a microseismic monitoring study. Thus, not only does the main theme lack a sound theoretical foundation and rely on hypothetical assumptions, but the microseismic monitoring work—although potentially worthy of a separate study—is only loosely related and is presented at a purely technical note level. Despite a limited connection, these are conceptually distinct topics that should be addressed separately.**

**Response:** We deeply appreciate the reviewer's thorough evaluation. To address this core concern:

We emphasize that microseismic monitoring provides critical input parameters (fracture angle α, span L) for the stress estimation model (Section 2.1)

Revised the structure to better articulate this methodological linkage

Added a conceptual framework diagram showing the data flow between components

Streamlined conclusions to focus on how microseismic results validate and enhance the force-source model

These changes demonstrate the integrated nature of our approach while maintaining methodological rigor.

**Examples:
1. A subheading appears as: “1111111Stress Analysis of the F16 Reverse Fault and Phase Boundary Zone.”**

**2. There is insufficient spacing between tables/figures and the following paragraphs.**

**Response:** We thank the reviewer for catching these presentation issues. We have:

- Corrected all malformed headings
- Standardized all figure/table placements with proper spacing
- Conducted full formatting review per journal guidelines
- The production-ready manuscript now meets all stylistic requirements.
