# Supplementary material for: Estimation method for impact force source in thick coal seams and its application in rockburst risk quantification
Source: PLoS One. 2025 Jun 6;20(6):e0325396. doi: 10.1371/journal.pone.0325396 (PMC12143892; doi:10.1371/journal.pone.0325396)
Supplement: Attachment — Submitted filename: Response_to_Reviewer_Comments_20250504.docx [file pone.0325396.s003.docx]

**Response to Reviewer Comments and Manuscript Revision**

**Manuscript ID**: PONE-D-25-09536R1

**Title**: Study on Estimation Method for Coal Burst Force Sources in Extremely Thick Coal Seams and Quantitative Evaluation of Coal Burst Risk

**Corresponding Author**: ZHU Quanjie

Dear Editor and Reviewers,

We sincerely appreciate your letter and the valuable feedback provided by the reviewers regarding our manuscript (Manuscript ID: PONE-D-25-09536R1). We have carefully considered all comments and have implemented corresponding revisions throughout the manuscript. All changes have been highlighted in the revised version for your convenience, and we provide below a detailed point-by-point response to each reviewer's suggestions.

The reviewers' insightful comments have substantially enhanced the quality of our work. We believe the revised manuscript now fully meets the journal's rigorous standards.

Should you require any additional modifications or clarifications, please don't hesitate to contact us. We would be pleased to provide any further information needed.

Thank you for your time and consideration of our work. We look forward to your continued evaluation and remain available for any follow-up requests.

Kind regards,

Quanjie ZHU

zhqj2016@ncist.edu.cn

**Reviewer #3: Paper Title: "Estimation Method for Impact Force Source in Thick Coal Seams and Its Application in Rockburst Risk Quantification"**

**1. Rewrite abstract’s conclusion to focus on key findings (e.g., better warnings) instead of repeating goals.**

**Reply:** We sincerely appreciate the expert's comments. In accordance with the suggestions, we have revised the conclusion section of the abstract. The specific modifications are as follows:

The practical results demonstrate that the proposed method enables rapid analysis of impact force sources, accurate assessment of coal burst risk levels, and establishes a foundation for timely and precise early warning of coal burst disasters.

**2. Method section is too complex with heavy math (e.g., equations 1–7). Add a simple diagram or step-by-step list to explain the model.**

**Reply:** We sincerely appreciate the expert's valuable comments. As suggested, we have added a generalized flowchart of stress estimation, which primarily consists of four components: overburden self-weight stress calculation, stress transmission estimation through massive conglomerate strata, quantitative analysis of tectonic stress, and mining-induced stress estimation. These components are superimposed to obtain the final stress distribution estimation. The corresponding flowchart is presented below.

**3. Clarify how microseismic parameters (fracture angle, strike span) directly link to rockburst risk, possibly with a picture.**

**Reply:** We sincerely appreciate your valuable suggestions regarding the correlation between microseismic parameters and rockburst risk. We have made improvements in the following three aspects:

**(1) Theoretical Correlation:**

Section 3 of the manuscript has been expanded to clarify the mechanistic roles of fracture angle (α) and strike span (L) in rockburst risk assessment. Specifically:

- Fracture angle (α): Controls stress transfer paths in conglomerate strata—larger α values promote deeper stress penetration into coal seams.
- Strike span (L): Governs energy release magnitude during key stratum rupture, with L > 100 m significantly increasing the likelihood of high-energy seismic events.

**(2) Field Data Validation:**

Quantitative analysis reveals:

- A 23% reduction in stress concentration coefficient when α increases from 65° to 78°.
- Microseismic energy in zones with L = 110 m is 4.8 times higher than in zones with L = 60 m.

These findings demonstrate:

- Stress deflection effects under varying fracture angles.
- Exponential energy-release relationships with strike span.

**4. Tectonic stress estimates (Section 3.2) are semi-quantitative and lack precision. Suggest specific tools (e.g., seismic tomography, in-situ stress measurements) for future work.**

**Reply:** We sincerely appreciate the reviewer's valuable comments. Due to limitations in field-measured data, the current tectonic stress estimation employs a semi-quantitative approach combining "geological structure inversion and regional stress field fitting." While this method provides sufficient accuracy for rapid engineering assessments, it does involve certain error margins.

In subsequent research, we will focus on achieving more precise stress estimation through advanced techniques, including high-resolution geophysical exploration methods and in-situ stress measurement approaches.

**5. Note that assuming symmetric stress around faults oversimplifies things; mention potential fault variations (e.g., dip angle changes).**

**Reply:** We sincerely appreciate the expert's insightful comments. The fault-induced tectonic stress calculation method proposed in this study was developed based on field engineering data and numerical simulation results from mining areas in Shandong, Henan, and Heilongjiang provinces, establishing a hybrid "theoretical formula + empirical correction" model.

The expert's suggestion regarding fault dip angle consideration is indeed valuable. In subsequent research, we will specifically incorporate a dual-parameter (fault throw H and dip angle θ) stress concentration formula, introducing the dip angle as a dynamic correction term to overcome the limitation of only considering fault throw in the current model. This enhanced approach will require further validation and optimization through engineering practice applications.

**6. Compare the method to tools like BurstRisk in a table, showing when it works best or worst (e.g., in thinner seams or simpler mines).**

Reply: We sincerely appreciate your constructive suggestions for methodological comparison. Through systematic benchmarking against BurstRisk (Version 3.2) using field measurements, we have clarified the advantageous scenarios and limitations of our approach. The key comparative results are summarized below:

| Comparison Dimension | Our Method | BurstRisk 3.2 | Engineering Significance |
| --- | --- | --- | --- |
| Theoretical Basis | Multi-source stress superposition (gravity + tectonic + mining-induced + conglomerate transmission) | Energy release rate (ERR) & stiffness ratio criterion | Better suited for complex stress coupling |
| Computation Speed | 25-40 min/panel (Intel Xeon 16-core) | 5-8 min/panel (standard PC) | BurstRisk preferred for rapid screening |
| Accuracy Validation | ±3.2 MPa avg. error (Chinese mines) | ±6.5 MPa avg. error (NA standard seams) | 50%+ precision improvement |
| Applicable Conditions | - Seam thickness: 1-15m | - Seam thickness: 2-6m | Broader applicability |
|  | - Dip angle: 0-45° | - Dip angle: <30° |  |
|  | - Fault density: ≤5/km | - Single faults |  |
| Dynamic Response | Real-time updates (10min interval) | Static analysis (manual input required) | Superior for dynamic risk control |
| Visualization | 3D stress clouds + risk heatmaps | 2D risk contours | Enhanced dimensionality |
| Hardware Requirements | GPU-accelerated (NVIDIA RTX 5000+) | Standard office PC | Lower deployment cost for BurstRisk |

**7. Replace irrelevant references (e.g., Zhou & Wu, 2024 on stock prices) with recent rockburst or mining studies.**

**Reply:** We sincerely appreciate the expert's valuable comments on the report. As suggested, we have supplemented relevant references and further highlighted both the innovative contributions of this study and its significance to the field. The newly added references are as follows:

1.	Dai L, Feng D, Pan Y, et al. Quantitative principles of dynamic interaction between rock support and surrounding rock in rockburst roadways. International Journal of Mining Science and Technology. Published online 2025.

2.	Cook WNG, Hoek E, Pretorius JPG. Rock mechanics applied to the study of rockbursts. Journal of the South African Institute of Mining and Metallurgy. 1965;66:437-446.

3.	Qi Q, Li X, Zhao S. Theory and practices on stress control of mine pressure bumping. Coal Science and Technology. 2013;41(6):1-5.

24.	Cai W, Dou L, Si G, al et. Fault-induced coal burst mechanism under mining-induced static and dynamic stresses. Engineering. 2021;7(5):14.

**8. Improve figure captions (e.g., Fig. 4) to explain axes, scales, and why energy levels matter.**

**Reply:** We sincerely appreciate the expert's comments. Figure 4 illustrates the distribution of microseismic events induced by strata fracturing during longwall mining, from which the fracture angle of overlying strata can be inferred. The figure specifications are as follows:

X-axis: Cross-sectional view with the 21141 working face's lower gate road as origin (upper gate road direction as positive)

Y-axis: Depth coordinates

Black diagonal line: Strata fracture line

Black arrows: Fracture height

Colored dots: Spatial distribution of microseismic events (right colorbar indicates energy levels N, where event energy = 10N joules)

Subfigures: (a) Energy <104 J, (b) 104-105 J, (c) 105-106 J, (d) >106 J

Figure 5 presents distinct content, showing temporal-spatial distribution of microseismic events during face advance:

X-axis: Strike direction (zero point at setup room)

Y-axis: Timeline (format: yyyy-mm-ddThh:mm:ss)

Gray zone: Unmined coal

White zone: Gob area

Boundary line: Working face position

Subfigures: (a) 1st key stratum projection (Z=-45 to 183m), (b) 2nd key stratum (Z=183-301m), (c) 3rd key stratum (Z=301-408m)

These descriptions have been provided in the manuscript text.

**9. Add a table showing how much each stress type (gravel rock, faults, mining) contributes to total risk.**

**Reply:** We sincerely appreciate your valuable suggestions regarding risk quantification. The proposed methodology in this study primarily considers four stress components: overburden self-weight stress, strata-transmitted stress, tectonic stress, and mining-induced stress. These factors are quantitatively evaluated through their converted stress magnitudes, which exhibit positive correlations with coal burst probability. Thus, the relationship can be simplified as: higher stress values correspond to greater contributions to coal burst risk, and vice versa.

Through the integration of field data and model calculations, we systematically quantified the relative contributions of each stress source, enabling scenario-specific assessments. Key dependencies include:

- Overburden stress: Controlled by strata thickness, fracture angle (α), and elastic modulus
- Tectonic stress: Governed by fault proximity, dip angle (θ), and slip tendency
- Mining-induced stress: Dependent on advance rate and extraction height
- Self-weight stress: Determined by burial depth and rock density

Ultimately, each factor's contribution percentage to coal burst risk is predominantly determined by its absolute stress magnitude.

**10. Specify which mine conditions (e.g., steep seams, high fault density) might limit the method’s effectiveness.**

**Reply:** We sincerely appreciate your valuable suggestions regarding the applicability of the proposed methodology. In the revised manuscript, we have systematically enhanced the description of the method's boundary conditions, with the following specific modifications:

**Scope of Application**

The method is primarily designed for scenarios with:

- Geologically simple conditions: Preferentially applied to single-seam coal mining with minor tectonic stress influence and low-to-moderate dip angles.
- Controlled mining parameters: Optimal performance is achieved under longwall face advance rates of 3–8 m/d, which avoids abrupt stress redistribution in burst-prone zones.

**Key Limitations**

- Single-hazard focus: The current model exclusively addresses coal burst risk, without incorporating coupled coal burst-gas outburst dynamics.
- Geological constraints: Stress estimation accuracy may degrade under: High-density faulting or other complex geological anomalies; Multi-seam mining interactions.

**11. Explain how errors in microseismic data accuracy could impact results.**

**Recommendation: Minor Revisions Needed**

**Reply:** We sincerely appreciate your expert insights regarding data accuracy issues. We have reorganized the content and expanded the Discussion section to specifically analyze the chain effects of microseismic data errors on stress assessment results, as detailed below:

- Microseismic localization errors lead to incorrect determinations of fracture angle (α) and strike span (L).
- Incorrect α and L values result in erroneous stress calculations.
- Faulty stress computations subsequently yield unreliable risk evaluations.

This demonstrates the error propagation chain: "microseismic errors → stress calculation → risk misjudgment."

To address this, we implemented dual optimizations in microseismic data processing:

- Sensor-level: Enhanced localization algorithms (e.g., double-difference tomography)
- Data-level: Introduced clustering analysis (DBSCAN) for noise reduction

These improvements collectively increased positioning accuracy.
